# National taxation on sugar-sweetened beverages: a scoping review and time series analysis

**DOI:** 10.1093/eurpub/ckac129.459

**Published:** 2022-10-25

**Authors:** L Villani, M Sassano, C Castagna, G Quaranta, R Pastorino, S Boccia

**Affiliations:** 1 Section of Hygiene, Università Cattolica del Sacro Cuore, Rome, Italy; 2 Department of Medical and Surgical Sciences, Università di Bologna, Rome, Italy; 3 Department of Woman and Child, Fondazione Policlinico Universitario A. Gemelli, Rome, Italy

## Abstract

**Background:**

The intake of Sugar-Sweetened Beverages (SSBs) has increased around the world, leading to a growing burden of disease due to these beverages, such as obesity, diabetes, and heart disease. Taxation is a major action for comprehensive programmes aimed at reducing consumption of sugars. For these reasons, the aims of our study were to systematically summarize national tax legislation on SSBs and to assess the impact of these laws on the prevalence of overweight, obesity, and diabetes.

**Methods:**

We conducted a scoping review to summarize the landscape of national tax laws on SSBs implemented worldwide. We included any document reporting both currently into force and past national tax laws addressing SSBs. As to the time series analysis, data regarding the national prevalence of obesity, overweight, and diabetes were retrieved from WHO Global Health Observatory data repository.

**Results:**

As of July 2020, 34 countries worldwide implemented SSB taxation (amount-specific and ad valorem tax design), of which 17 (50.0%) in high-income countries, 12 (35.3%) in upper-middle income countries, and 5 (14.7%) in low-income countries. As for overweight, Hungary was the only country showing a slower rate of change after the taxation. Regarding obesity, France, Guatemala, Hungary and Panama showed a deceleration of the rates of change after the intervention. Eventually, Hungary and Tonga exhibited a one-time decrease of diabetes prevalence at the intervention point. Decelerating rates of change in the post-intervention period was also found for Guatemala and Fiji.

**Conclusions:**

Laws targeting SSBs showed, at least in part, to be an effective measure to reduce the prevalence of overweight, obesity, and diabetes. Less than one fifth of worldwide countries have implemented national taxation policies. Finally, while taxation might be effective to reduce SSB consumption, other types of Public Health interventions, such as educational initiatives, should not be neglected.

**Key messages:**

• Sugar-Sweetened Beverages (SSBs) are associated with obesity, diabetes, and heart disease. Taxation can be an important tool to reduce the consumption of SSBs.

• Prevalence of obesity, overweight and diabetes decreased in countries that adopted taxation. However, other strategies such as educational programs should be implemented to reduce the intake of SSBs.

